# Malignant epignathus teratoma

**DOI:** 10.2349/biij.4.2.e18

**Published:** 2008-04-01

**Authors:** SC Too, S Ahmad Sarji, YI Yik, TM Ramanujam

**Affiliations:** 1 Department of Biomedical Imaging, Faculty of Medicine, University of Malaya, Kuala Lumpur, Malaysia; 2 Department of Surgery, Faculty of Medicine, University of Malaya, Kuala Lumpur, Malaysia

**Keywords:** Epignathus teratoma, imaging features

## Abstract

A baby boy who had a left facial mass detected on antenatal ultrasound was delivered by Caesarian section after foetal distress was detected. Imaging investigations by plain radiographs and MRI showed a large mass with calcifications, soft tissue, fat and fluid components. A total surgical excision was perfomed and histology examination showed teratoma with no malignant features. Two weeks postoperatively, there was rapid recurrence of the tumour with intracranial involvement and obstructive hydrocephalus shown on MRI. The tumour was inoperable at surgery and the baby subsequently died at 5 weeks of life. This case describes the clinical course and imaging features of a neonatal epignathus teratoma with malignant and aggressive features.

## INTRODUCTION

Head and neck teratomas are rare congenital tumours, usually benign in nature. They are usually of enormous size and commonly cause respiratory compromise to the upper airway. The term ‘epignathus tumour’ refers to teratomas of the oropharyngeal cavity in neonates without specifying the site of origin [1]. Epignathus tumours are extremely rare head and neck teratomas, which are not known to be malignant but have the potential to extend into the cranium and involve the brain [2]. This case study describes a case of a large epignathus teratoma with malignant and aggressive features clinically and on imaging. There were high levels of serum α-fetoprotein (AFP) and β -human chorionic gonadotropin (β hCG), and an MRI demonstrated rapid recurrence of the tumour after resection.

## CASE REPORT

A baby boy was born to a 32-year-old primigravida at 34 weeks gestation. Antenatal ultrasound done at 28 weeks detected polyhydramnios and foetal anomaly, where a huge mass of mixed cystic and solid echogenicity was detected arising from the left side of his face, measuring more than 10 cm in its maximum dimension. The baby was born by Caesarian section after foetal distress was detected. He developed respiratory distress at birth which required intubation and assisted ventilation.

**Figure 1:**
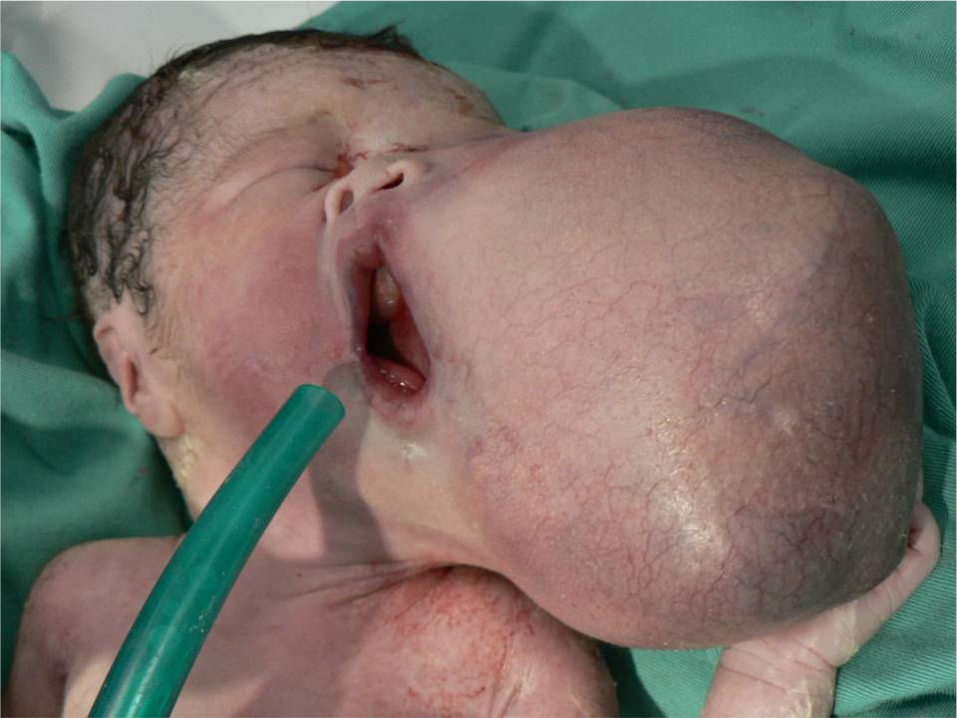
Photograph of the baby immediately after delivery showing a large left facial mass distorting the external facial anatomy with displacement of the nose and left eye.

Physical examination revealed a large spherical mass at the left cheek measuring approximately 8 cm x 10 cm in size. There was significant displacement of his mouth to the right, his nose superiorly and his left eye superolaterally, due to the sheer size of the mass (Figure 1). The overlying skin appeared healthy, with no ulceration or discolouration. On superficial palpation, the mass had a mixed soft to hard consistency. There was a haemangioma measuring 3 cm x 1 cm in size over his right nipple. Further examination showed that the other systems were normal. Maturity scoring of the baby was appropriate for age. The differential diagnoses from physical examination included teratoma, primitive neuroectodermal tumour (PNET) and embryonal sarcoma.

**Figure 2:**
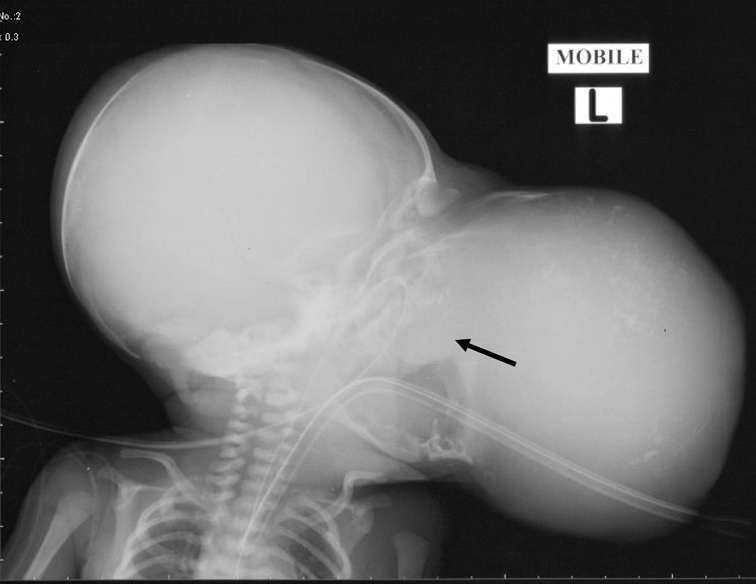
Plain radiograph of the head showing a large soft tissue mass protruding from the anterior aspect of the face with calcifications within it. Note the under-developed left temporomandibular joint (black arrow).

**Figure 3:**
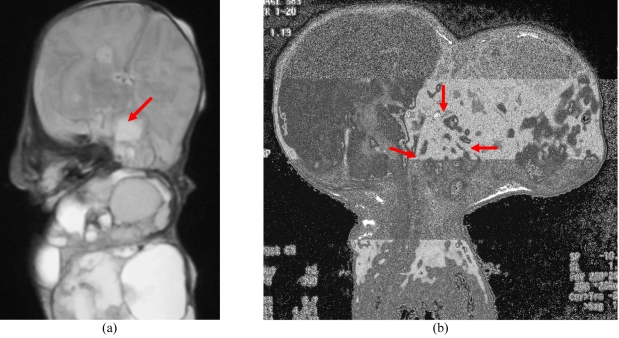
MR images: (a) T2 weighted ( TR 4500, TE 94) sequence in coronal plane showing the external portion of the tumour consisting of lobulated well defined areas of fluid, solid tissue and fat, in keeping with teratoma. There is involvement of the left temporal lobe of the brain (red arrow); (b) T1 weighted (TR 9.7, TE 4.1) contrast enhanced sequence in sagittal plane. Internally, the tumour is occupying the oropharynx, nasopharynx and oral cavity, effacing normal anatomy (red arrows).

Haemoglobin, white blood cell count and platelets were normal. Serum α-fetoprotein (AFP) measured 56852 IU (normal 0-8 IU) and β-human chorionic gonadotropin (β hCG) 14 IU (normal <10 mIU). The high levels were in keeping with a teratoma. A plain radiograph of the face and skull showed a wide opened oral cavity, poorly developed left temporomandibular joint and calcifications within the large soft tissue density mass (Figure 2). On MRI, there was a large and lobulated tumour that demonstrated mixed signal intensities in keeping with fluid, fat and soft tissue components on both T1 and T2 weighted sequences. The left globe of the eye was displaced superiorly. The internal portion of the tumour effaced all normal anatomy whereby the left side of the oral cavity, oropharynx, nasopharynx, palate and left temporomandibular area had no clear margins from the tumour. There was extension of the tumour into the left temporal fossa (Figure 3).

Surgery on day 7 of life revealed findings which largely corresponded to findings on MRI. The tumour extended from the left temporomandibular joint to the nasal septum. It occupied the oropharynx and nasopharynx, caused elevation of the buccal mucosa and upper airway obstruction. There was extension superiorly into the base of the skull and inferiorly into the oral cavity, displacing the left mandible, as well as posteriorly to the lesser wing of the sphenoid where there was a defect covered by a thin membrane. The condyle and coronoid process of the left mandible were not well developed and no articulation existed between the left mandibular condyle and the temporal bone. The tumour was removed en-bloc with minimal difficulty and the cavity packed with surgicel and gelfoam. A skin flap was raised via circumferential dissection to cover the incision site over the left side of the face.

**Figure 4:**
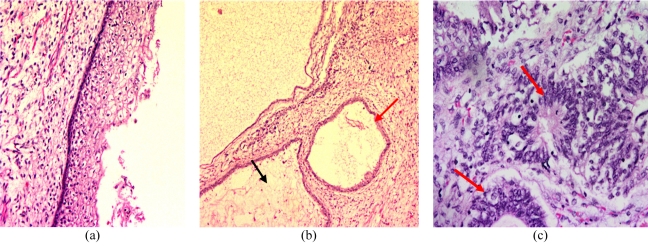
Histopathology of the tumour tissue consisting of a haphazard mixture of organoid mature tissue: (a) skin adnexal tissue (Haematoxylin-eosin (HE) stain, x 10); (b) adipose cells (black arrow) and cysts lined by stratified squamous epithelium (red arrow) (HE stain, x 4); (c) primitive neuroepithelial cells arranged as rosettes (red arrows) (HE stain, x20).

Cut section of the tumour showed multi-loculated cysts containing serous fluid and solid areas. Histopathology revealed tumour tissue consisting of a haphazard mixture of organoid mature tissue composed of skin adnexal tissue and microtubular structures. Calcifications and cysts lined by stratified squamous epithelium with rosettes were identified in one area of the section. These features were in keeping with a teratoma with immature neural elements. No mitotic or undifferentiated elements were seen (Figure 4).

**Figure 5:**
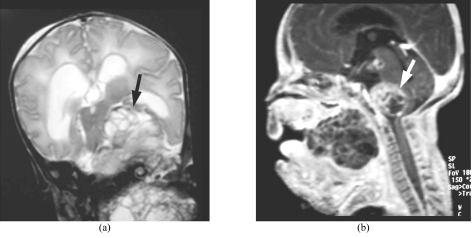
MR images two weeks after removal of the teratoma: (a) T2 weighted (TR 4500, TE 94) in coronal plane; and (b) T1-weighted (TR 9.7, TE 4.1) contrast-enhanced sequence in sagittal plane, showing recurrence. Tumour is seen occupying the oral cavity with extension into the left temporal fossa (black arrow) and brain stem compression (white arrow). There is evidence of moderate hydrocephalus.

Post-operatively, there was a significant decrease in serum α-fetoprotein to 4408 IU/ml. However, the left side of the face became increasingly swollen and there was difficulty extubating the baby despite clearance of tumour from the airway at surgery. A repeat MRI and CT scan done 2 weeks after surgery showed a mass of solid and cystic nature occupying the oral cavity with extension into the left temporal fossa. It showed avid enhancement with contrast and it extended posteriorly compressing the brain stem and causing obstructive hydrocephalus (Figure 5). The endotracheal tube was displaced to the right side by the mass. The baby underwent another surgery. It showed recurrence of tumour in the oral cavity and oropharynx with extension into the left temporal fossa through the membranous covering. There was no further attempt to excise the tumour. A tracheostomy and external ventricular drainage was planned, but the baby succumbed 3 weeks later.

## DISCUSSION

In Greek, teratoma means “monsterous tumour”. It is a tumour composed of multiple tissues foreign to the normal organ in which it arises. These tissues contain all three primordial germ cell layers (ectoderm, mesoderm and endoderm) [1, 2, 3, 4] and histologically they may be mature, immature or malignant. Teratomas occur with an incidence of 1:4000 live births [2]. They may arise from different sites of the body; the most common site in the newborn is the sacrococcygeal region, accounting for nearly 40% of the total cases [5]. Other sites include the gonads, head and neck, mediastinum, retroperitoneum, brain, spinal cord and liver. Less than 5% occur in the head and neck. Facial involvement occurs in 1.6% [5]. Epignathus are teratomas arising from the soft or hard palate in the region of the Rathkes pouch or in the nasopharynx in the region of the basisphenoid, tongue, sinuses, mandible or tonsil. Development may be from the midline or lateral to midline [2]. When large, they fill the oral cavity and can protrude externally, distort facial anatomy and often cause respiratory embarrassment at birth. Malignant degeneration of teratomas is estimated to be between 5% and 30% [5]. Malignant epignathus teratomas are rare and there have been no recent reports of it. After complete excision, these tumours do not recur. Serial measurement of serum α-fetoprotein (AFP) is necessary as failure of the expected gradual fall of this marker postoperatively may indicate the existence of residual malignant elements or development of metastasis. In malignant teratomas, AFP levels are drastically raised and β-human chorionic gonadotropin (β hCG) may occasionally be secreted [5]. Both markers were significantly raised in our patient, suggesting a malignant teratoma. The histopathology did not reveal any malignant features although immature neural elements were present. Overall, neuroectodermal elements (both mature and immature) frequently dominate in childhood teratomas and are found more commonly in teratomas of the head and neck region. Childhood teratomas with neuroectodermal elements may be confused with PNETs at histologic examination if there is inadequate tissue sampling. More importantly, the presence of some immature neural tissue associated with a teratoma in a young child does not reduce the prognosis.

Teratomas can be detected early with antenatal ultrasound. MRI has the advantage of not utilising ionising radiation and antenatal MRI diagnosis of teratoma in the foetus has been described [4, 6]. Ultrafast MRI techniques for antenatal diagnosis and characterisation of cervicofacial masses using echo-planar imaging and half-fourier single-shot turbospin-echo sequences to minimise foetal motion and breathing artifacts has been described by Kenneth W. Liechty et al. [6]. This may be a useful modality for diagnosis once the mass is revealed by antenatal ultrasound examination. In the neonatal period, both CT and MRI have been descibed as useful for complete assessment of the tumour, to determine its relationship to surrounding structures, its extension and any complications caused by the tumour [3, 4]. In institutions with access to MR imaging, this modality may add more information with regard to diagnosis and presurgical assessment than CT is able to provide. MRI enables characterisation of the components of the teratoma. The high fat content of teratomas is the cause of their strong signal on T1-weighted images and allows their differentiation from cystic hygromas, which are less intense on T1- weighted images but become more intense on T2-weighted images [3]. The first preoperative MRI scan in this case proved invaluable in diagnosis and preoperative assessment of the teratoma. The second MRI scan showed rapid and aggressive recurrence of tumour as well as complication of its extension and compression of vital structures. However, MRI has the disadvantage of requiring long scanning time. As CT scanning better demonstrates bony defects, this modality may be needed as an adjunct to MRI in cases in which intracranial communication is suspected but not demonstrated on MRI [4]. Advantage of CT is that it provides rapid assessment and fast multiplanar reconstruction, enabling accurate assessment of bone involvement. It also establishes the presence of calcifications, which are present in 50% of teratomas [3]. In our case, plain radiography of the head revealed calcifications in the tumour.

Epignathi involving and destroying brain tissue has been described and is associated with poor prognosis [2]. Exclusion of intracranial extension is an important part of preoperative management. An intracranial extension must be suspected in the event of sphenoid dehiscence [1]. In this case, intracranial extension was evident on the first MRI. The components of the left temporomandibular joint were not identified on imaging. Intra-operative findings revealed a defect of the lesser wing of sphenoid that was covered with a membrane and absence of articulation between a poorly developed left mandibular condyle and the temporal bone.

Mortality rate associated with large teratomas in the head and neck are generally high in the absence of a well-prepared resuscitation team or meticulous delivery planning to secure the airway, as the majority of these teratomas are associated with obstruction of airway and difficulty in intubation. In the patient described here, marked distortion of facial anatomy with involvement of the nasopharynx and oropharynx as well as extension into the posterior pharynx led to initial respiratory distress. Subsequently, brain stem compression by recurrent tumour accounted for respiratory difficulties.

Ninety percent of neonatal teratomas are benign and treatment is primarily by surgical removal [5]. In the absence of intracranial extension, radical treatment of epignathus teratoma consists of early and complete surgical resection. These tumours are generally encapsulated or pseudoencapsulated and complete resection can be achieved without sacrifice of any vital structures despite alarming appearances on imaging [4]. In our case, the tumour was easily removed despite its large size and effacement of anatomy of the base of skull structures. However, due to its rapid recurrence and aggressive nature, it is likely that this was a rare case of malignant epignathus teratoma.

In conclusion, we describe a rare case of epignathus teratoma showing malignant features clinically and on imaging. The case proved to be a great challenge for all disciplines involved in management of the baby.
